# Do We Need to Put Society First? The Potential for Tragedy in Antimicrobial Resistance

**DOI:** 10.1371/journal.pmed.0030029

**Published:** 2006-01-10

**Authors:** Kevin R Foster, Hajo Grundmann

**Affiliations:** 1 Kevin R. Foster and Hajo Grundmann wrote this article while at Wissenschaftskolleg zu Berlin (Institute for Advanced Study), Berlin, Germany. Kevin R. Foster is now at the Department of Biological and Environmental Sciences, University of Helsinki, Finland, and Hajo Grundmann is also at the National Institute for Public Health and the Environment, Bilthoven, The Netherlands, and the Department of Microbiology, University Medical Center, Groningen, The Netherlands.

## Abstract

Antimicrobial use presents a dilemma, say Foster and Grundmann. Appropriate use can benefit individual patients but carries a cost to society by selecting for resistant strains that are difficult to treat.

The use of antimicrobials has caused a proliferation of resistant pathogens (Figure 1), and most worryingly, some bacterial strains are resistant to multiple classes of drugs [1,2]. Policies are now being implemented to reduce antimicrobial use, with some encouraging successes [3,4]. However, here we argue that current policies may only partly solve the problem. In particular, they do not address the conundrum at the heart of antimicrobial resistance: the solution may ultimately require us to put society before the individual. That is, halting the rise of resistance may only be achievable if some patients go untreated. We defend this uncomfortable conclusion using the logic of the well-known social dilemma “the tragedy of the commons” [5]. More data on the societal costs of resistance are required to evaluate the potential for a tragedy of antimicrobial resistance and the moral dilemma that it would present.

**Figure 1:**
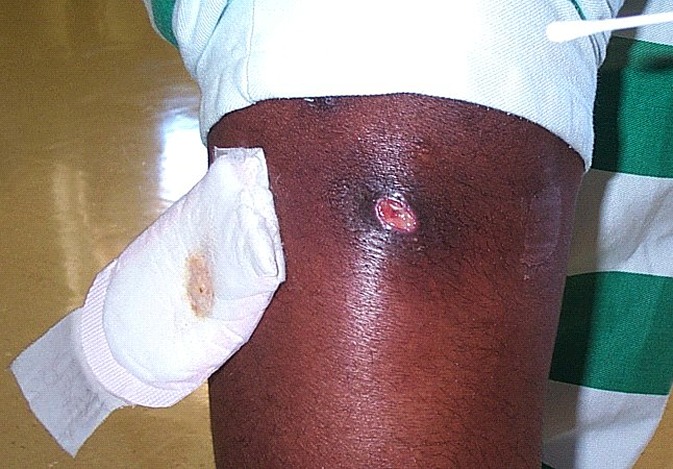
A Cutaneous Knee Abscess Caused by MRSA MRSA is resistant to many common antibiotics, making it difficult to treat. (Photo: CDC/Bruno Coignard/Jeff Hageman)

In the late 19th century, pioneering microbiologists laid the foundations of germ theory, which became one of the most powerful explanations for epidemic disease [6,7]. It was quickly understood that chemical substances that kill microbes could defeat infectious diseases. In the middle of the last century, an apparently endless stream of newly developed antimicrobial compounds, most famously penicillin, left the impression that humanity had established superiority over the microbial world once and for all [7]. But it has since emerged that this is far from the truth [3,7,8]. For decades, we have created an environment where any pathogens that can survive antimicrobial treatment have a strong selective advantage. The result has been the proliferation of resistant strains [6,7] and the origin of bacteria resistant to multiple antibiotic classes [1,2].

Antimicrobial chemicals are frequently used where they are not needed. For example, antibacterials are often prescribed for viral infections [3,4,8] and the widespread availability of over-the-counter antimicrobials in many countries can result in ineffective self-medication [8]. Large volumes of antimicrobials are also used in agriculture and veterinary medicine [9,10], and in many consumer products in which they do not always have a documented function [11]. The emergence of antimicrobial resistance, therefore, can be greatly slowed by reducing inappropriate antimicrobial use [4,12] and considerable efforts are currently underway to promote this goal. These include the development of guidelines [13], and educating physicians and the public about best practice [3,4,8]. Another priority is to develop improved diagnostic tools that allow rapid identification of pathogens and the appropriate antimicrobial treatment [12]. The pressing need for these programs is clear. However, here we argue that they do not address a conundrum that is central to the problem of resistance. Protecting the effectiveness of antimicrobials may only be possible if we put society before the individual.

## The Potential for Tragedy

Antimicrobial use presents a dilemma [14]. Appropriate use can benefit individual patients but carry a cost to societal health by selecting for resistant strains that are difficult to treat [15] (Figure 2). Baquero and Campos [16] recently argued that this dilemma mirrors what Hardin termed “the tragedy of the commons” [5,17–19]. Hardin's phrase refers to common land to which many people have rights. Every herdsman knows that putting too many cows upon a pasture will eventually destroy it by overgrazing. However, when pastures are a shared commons, the benefit of adding a cow goes entirely to the owner (the individual) but all herders share the cost (society). The rational solution for an individual is to keep adding cows, even though this leads to the deterioration and possible collapse of the pasture, at a large cost for all [5,17–19].

**Figure 2:**
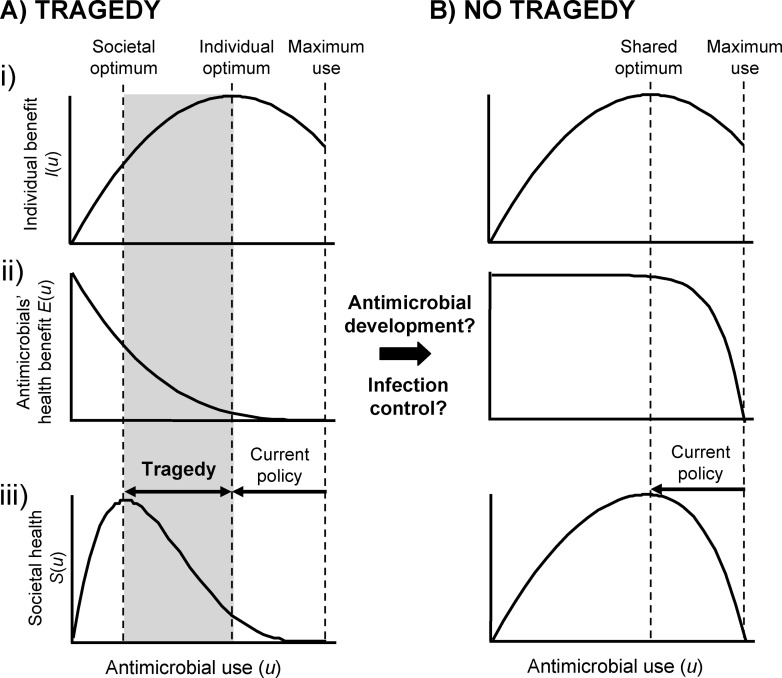
Is Antimicrobial Resistance a Tragedy of the Commons? (A) Tragedy scenario. A simple model that illustrates the potential for moral dilemma in antimicrobial use. Optimizing antimicrobial use *(u)* for the good of all requires consideration of both society and the individual patient. (i) Individual health benefit from antimicrobial use *I(u)*. (ii) Cost to societal health from decreased effectiveness of antimicrobials *E(u)* as a result of the evolution of antimicrobial-resistant pathogens. We assume that the societal benefit from reducing transmission rates is small and do not include it here. iii) Overall effect of antimicrobial use on societal health *S(u) = I(u)E(u)*. Ensuring that antimicrobials are only used for infections they can treat is a technical solution that will only take us to the individual optimum, which in this illustrative example is far from the real optimum. (B) Best-case scenario. Low to moderate antimicrobial use has little impact on our ability to treat later infections and is only weakly costly to societal health *E(u)*. This means there is no tragedy. The true nature of *E(u)* is unknown but it is a function of investment in both new antimicrobials and infection control, which may be able to shift us from scenario A to scenario B.

Hardin applied this analogy to the problems of overpopulation, shared fisheries, and taxation [5]. Baquero and Campos [16] have argued that the similarity to the problem of antimicrobial resistance means that we can make use of reputation effects to limit antimicrobial prescription (i.e. if overprescription is seen as damaging to the reputation of the doctors), as discussed below. What is most important for our discussion, however, is Hardin's key insight that a tragedy of the commons lacks a technical solution, which he defined as “one that requires a change only in the techniques of the natural sciences, demanding little or nothing in the way of change in human values or ideas of morality.” This insight is important because the current campaign to ensure that antimicrobials are only used where they will work is such a technical solution. This campaign is very important and will help to slow the evolution of resistance, but Hardin's argument indicates that we may need to go further. Protecting the antimicrobial commons, and hence the collective best interest, may require society sometimes to act against an individual patient's best interests (Figure 2A).

Clearly, any policy that acts against a patient's interest should be a last resort and would raise serious ethical concerns that need careful consideration [14,20]. That said, the importance of restricting diagnostic and therapeutic options to patients is already well understood by general practitioners who are increasingly obliged to divide medical resources among patients along firm budget lines [18,21]. The unfortunate reality is that individuals do not always receive the full extent of the treatment that they desire.

But what would putting society first mean for antimicrobial use? This is not yet clear. In the best-case scenario, the individual and societal optima for antimicrobial use will turn out to be similar, and the current focus on stopping inappropriate use [3,4,8] will indeed be sufficient (Figure 2B). However, if it emerges that what is good for society is markedly different to what is good for the individual, then society will benefit from reductions in use beyond those currently planned (Figure 2A). That is, society will benefit from further reductions in the number of times that each patient takes a course of antimicrobials in order to limit evolutionary selection for resistant strains. Such reductions might include severe limits on the use of new and broad-spectrum antimicrobials [14], or leaving milder, mostly self-limiting bacterial infections untreated. In the extreme case that we face a complete loss of antimicrobial effectiveness, some antimicrobials might be reserved only for dangerous and potentially life threatening infections.

## Is Antimicrobial Use a Tragedy of the Commons?

Understanding just how far antimicrobial use should be restricted is a major challenge for the future. The problem is that the optimal solution of a tragedy of the commons requires a clear idea of the relative costs and benefits to both the individual and society [17] (Figure 2), and it is here that large gaps in our knowledge exist [15,22]. While we have an idea of what the cost to a patient is for leaving an infection untreated, we need to better understand the benefits, which are likely to include leaving gut flora unharmed [8,13] and reducing the risk of resistance in later infections [4,8,23].

More data on the societal effects of antimicrobial use are also required to understand the potential for a tragedy of antimicrobial resistance [15,22]. Antimicrobial use by a patient can benefit others by preventing the pathogen being passed on but also carries the cost of promoting resistance [6,7]. And while it is clear that antimicrobial use increases the frequency of resistant strains that cause casualties [2], the full impact of resistance upon society is still poorly understood [15,22]. Although challenging, attempts to assess the societal costs of resistance will be helped by the strong differences in antimicrobial use between countries [24], which means that the effects of resistance can be monitored in communities with differing levels of use (Figure 2Aii). Agricultural studies may also provide valuable data, because a strategy that leaves animals untreated raises fewer ethical concerns than an equivalent strategy in our own society. The case for strong reductions in human antimicrobial use would be strengthened by evidence from agriculture that such reductions greatly prolong their effectiveness. Furthermore, any reductions in agricultural antimicrobials may have a knock-on benefit in reducing the incidence of resistant strains in human infections [9,10].

A better understanding of the costs and benefits of antimicrobial use, therefore, is a highly desirable goal for future research. However, it should also be emphasized that public policy can affect the severity of societal costs and the basis for any tragedy of antimicrobial resistance (Figure 2). Until now, we have been able to avoid many health effects of resistance through the development of new antimicrobial compounds [1,2]. Unfortunately, development by private firms is decreasing rapidly as the discovery of new compounds becomes more and more challenging [1], and consequently, financially costly. And these costs are set to increase as the campaigns to limit antimicrobial use further reduce profits from the sales of new compounds [14]. The impact of resistant strains on society, therefore, can be reduced by policies that promote antimicrobial development such as government investment in public-private partnerships [2,25] and the careful use of patents [26–28].

Another strategy that can reduce the health impact of resistant pathogens is hospital infection control, where a resistant pathogen is carefully monitored and targeted for special contact isolation and decontamination measures in a “search and destroy” policy [29–31]. This strategy has resulted in some notable successes with outbreaks of methicillin-resistant Staphylococcus aureus (MRSA) [31] (see Figure 1) and vancomycin-resistant enterococci [29], but it cannot contain the spread of resistance outside of institutions and, like development of new drugs, the strategy comes at considerable economic cost [32,33]. So while it is clear that investment in development and infection control will play an important role in reducing the health impact of resistance (Figure 2Aii), it is less clear that these strategies will eliminate the basis for tragedy altogether. We may, therefore, have to face up to the reality of a tragedy of antimicrobial resistance (Figure 2A).

## Could Further Restrictions in Antimicrobial Use Be Achieved?

The recent campaigns to discourage antibiotic use for common colds and to limit antibacterials to bacterial infections have met with success, but they also underline the difficulty in changing society's attitudes and behavior [3,4]. If further restrictions were deemed appropriate, could these be achieved? Here we might again turn to Hardin, who proposed two candidate solutions to a tragedy of the commons: “mutual coercion mutually agreed upon,” and privatization. However uncomfortable, in the event that antimicrobial use must be further restricted, both might play a role.

Coercion in society frequently takes the form of taxation, such as the use of parking fees when space is limited [5]. Similarly, prescribers or patients might be offered the choice of paying an antimicrobial-use levy or instead using alternative remedies. Coercion might also be achieved through new government regulations, which have already proved effective at reducing antimicrobial use in several countries [34]. More local regulation can be achieved by exploiting the preexisting management structures that exist in many health care settings to ensure that medical resources are divided equally [8,21]. Privatization solves the tragedy of the commons by dividing up the resource so that costs from selfishness feed back directly upon the individual owner [5]. But antimicrobial effectiveness cannot be divided in this way, making true privatization impossible. However, by analogy, careful tracking of antimicrobial prescriptions would create a feedback that enables individuals to be held accountable for extremes of use.

Hardin's solutions to a tragedy of the commons assume that humans behave as selfish, rational individuals who—if unmanaged—will display no regard for the interests of society. Although there is no doubt that humans are capable of selfishness, this assumption of rational “Homo economicus” behavior is being increasingly challenged [19,35–37]. For example, increased cooperativity is predicted whenever selfishness is damaging to reputation [19,37], and Baquero and Campos have argued that antimicrobial use will be decreased if we can establish a context in which overprescription is damaging to the reputation of doctors [16].

In addition, studies in which participants are asked to divide up shared resources show that humans behave less selfishly than simplistic self-interested strategies predict. This highlights the importance of human norms for cooperation [19,36]. If these norms translate to health care decisions (although it is not certain that they will), then educating patients about societal benefits will help decrease antimicrobial use [4]. The power of any societal argument is likely to be greatest when benefits accrue on a local scale [14]. Local benefits are realistic given the strong regional effects of differences in antimicrobial use and resistance, which suggest that reducing antimicrobial use can benefit a region or nation even if its neighbors adopt a less effective program [38].

## Conclusion

Hardin's tragedy of the commons has proved to be a powerful analogy for understanding the problem of protecting the benefit we all receive from public goods [5,17–19,36]. It finds particular relevance in the growing crisis of antimicrobial resistance, where use of antimicrobials threatens to undermine the protection they provide to society as a whole. The questions of how far to reduce antimicrobial use, and at what cost to individual patients, represent a central unanswered problem in the battle against resistance (Figure 2). The answer requires a better understanding of the effects of antimicrobial use on both the individual patient and society as a whole. In particular, we need to better understand the societal costs of resistant pathogens and the potential for investments into new antimicrobials and infection control to limit these costs. These empirical challenges exist alongside the ethical question of whether we should ever resort to a strategy that leaves patients untreated. It is the challenging task of physicians, public health agencies and governments to evaluate the severity of the situation and decide what should be done. Perhaps the strongest message from Hardin's analogy is that difficult choices may lie ahead [5,18]. Solutions to a tragedy of the commons do not come easily and are likely to require brave policy decisions.

## Abbreviation

MRSA: methicillin-resistant Staphylococcus aureus
